# Synthesis, Structure and Biological Activity of 6-R_3_Si(Ge, Sn)-Substituted 5-Fluorouracils

**DOI:** 10.1155/MBD.1994.65

**Published:** 1994

**Authors:** E. Lukevics, L. Ignatovich, N. Shilina, A. Kemme, N. Sjakste

**Affiliations:** 1 Latvian Institute of Organic Synthesis 21 Aizkraukles Riga LV-1006 Latvia

## Abstract

Direct lithiation of 1-(2-tetrahydrofuryl)-5-fluorouracil (Ftorafur) has been investigated. The treatment of ftorafur with lithium diisopropylamide (LDA)at –70℃ in ether-hexane, followed by the reaction with various electrophiles afforded the corresponding 6-substituted silicon, germanium and tin derivatives of ftorafur. The results of biological investigation indicate the ability of germanium–modified nucleoside analogues to interfere with transcription and replication processes.


### SYNTHESIS, STRUCTURE AND BIOLOGICAL ACTIVITY OF 6-$R_3$Si(Ge, Sn)-SUBSTITUTED 5-FLUOROURACILS

E. Lukevics, L. Ignatovich, N. Shilina, A. Kemme and N. Sjakste

Latvian Institute of Organic Synthesis, 21 Aizkraukles, Riga, LV-1006 Latvia

### Abstract

Direct lithiation of 1-(2-tetrahydrofuryl)-5-fluorouracil (Ftorafur) has been investigated. The treatment of ftorafur with lithium diisopropylamide (LDA) at –70°C in ether-hexane, followed by the reaction with various electrophiles afforded the corresponding 6-substituted silicon, germanium and tin derivatives of ftorafur. The results of biological investigation indicate the ability of germanium-modified nucleoside analogues to interfere with transcription and replication processes.

### Introduction

The chemistry of nucleosides is a rapidly growing area of research. Nowadays the synthetic analogues of nucleosides are used as antimetabolites in biochemistry and medicine. Examples are antiviral agents such as the nucleoside analogues 3'-azidothymidine possessing anti-HIV activity and acycloguanosine with antiherpes activity, the antitumor agents Ftorafur and 5-fluorouridine, and "antisense-RNA" and "antisense-DNA" used as gene blockers.

Recently the lithiation of uracil acyclonucleosides was employed to obtain 6-substituted derivatives [1-3]. We have investigated the lithiation of another type of nucleoside analogues — N-1 — tetrahydrofuryl derivatives [4,5]. An antitumor agent 1-(2-tetrahydrofuryl)-5-fluorouracil (Ftorafur) [6] has been chosen for the introduction of organometallic substituent to C-6 position of uracil.

### Experimental

*Synthesis of 1-(2-tetrahydrofuryl)-5-fluoro-6-trimethylgermyluracil (**III**)*

n-Butyllithium (58.2 ml, 1.25 N in hexane) was added dropwise to a solution of freshly distilled i-$Pr_2NH$ (7.34 g, 0.073 mol) cooled to –10°C. After the addition of butyllithium the reaction mixture was stirred for 30 min then cooled to –78°C and dissolved in 100 ml of THF. After cooling a solution of ftorafur (3.4 g, 0.017 mol) in 70 ml of THF was added dropwise. The mixture was stirred for 1 h at –78°C, and a solution of trimethylchlorogermane (11.25 g, 0.073 mol) in 10 ml of THF was added dropwise at temperature kept below –70°C. The mixture was allowed to stand overnight. The solvents were removed *in vacuo*. The residue was dissolved in the saturated ammonium chloride solution in water and neutralized to pH 7 with the diluted HCl. After 1 h the desired product was filtered off (4.15 g, 77%). Compound **III** was recrystallized from EtOH, m.p. 132–134°C, on TLC it gave one spot ($CHCl_3$ : EtOH, 5:1, v/v, $R_f$ 0.70).

The compounds **I**, **II**, **IV** and **V** were prepared in the similar manner. Yields, melting points and analytical data are presented in Table 1.

Table 1. Analytical data of 6-substituted 1-(2-tetrahydrofuryl)-5-fluorouracils

| Compound | Yield (%) | M.p. (°C) | Formula | Analytical data (%) (found/calc.) | | |
|---|---|---|---|---|---|---|
| | | | | N | C | H |
| I | 2 | 162–3 | $C_{11}H_{17}FN_2O_3Si$ | 10.77/10.29 | 48.60/48.51 | 6.01/6.29 |
| II | 62 | 93–4 | $C_{14}H_{23}FN_2O_3Si$ | 8.94/8.91 | 53.85/53.48 | 7.39/7.37 |
| III | 77 | 132–4 | $C_{11}H_{17}FN_2O_3Ge$ | 8.72/8.84 | 41.90/41.70 | 5.30/5.41 |
| IV | 84 | 82–3 | $C_{14}H_{23}FN_2O_3Ge$ | 7.77/7.80 | 47.01/46.85 | 6.44/6.46 |
| V | 19 | 151–2 | $C_{11}H_{17}FN_2O_3Sn$ | 7.38/7.72 | 36.46/36.40 | 4.41/4.72 |

$^1$H NMR data were consistent with the structure of compounds I–IV

*Crystallographic Study*

The crystals of compounds I and III for X-ray measurements were grown from ethanol solution. The reflection intensities were collected on a Syntex-$P2_1$ single–crystal diffractometer using graphite–monochromated Mo-$K\alpha$ radiation ($\lambda$ = 0.71069 Å). The cell constants were obtained from a least–squares refinement on the setting angles of 20 reflections.

Crystal data: Compound I — $C_{11}H_{17}FN_2O_3Si$, M = 272.3, monoclinic, space group $P2_1/c$, a = 7.981(2), b = 11.067(3), c = 16.519(4) Å, $\beta$ = 115.77(2)°, V = 1313.9(7) Å$^3$, Z = 4, F(000) = 536, $D_x$ = 1.30 g/cm$^3$, $\mu$(Mo$K\alpha$) = 1.1 cm$^{-1}$.

Compound III — $C_{11}H_{17}FN_2O_3Ge$, M = 316.8, monoclinic, space group $P2_1/c$, a = 8.000(1), b = 11.1261(2), c = 16.598(3) Å, $\beta$ = 115.82(1)°, V = 1329.9(4) Å$^3$, Z = 4, F(000) = 608, $D_x$ = 1.50 g/cm$^3$, $\mu$(Mo$K\alpha$) = 13.0 cm$^{-1}$.

The data were collected at room temperature from a well–shaped colorless crystals with the $\omega/2\theta$ scan technique up to $2\theta_{max}$ = 40° for compound I and $2\theta_{max}$ = 55° — for III. No absorption correction was applied at measurement stage.

1351 reflections for I and 3070 reflections for III were collected, of which 958 and 2497 reflections were unique and found to have I > $2\sigma_1$.

The structures were solved by a direct method using program SHELXS [(7)]. The non–hydrogen atoms were found in a E-map. Initially, the positional parameters and isotropic temperature factors of all non–hydrogen atoms were refined by full–matrix least–squares procedure. At this stage an empirical absorption correction for compound III (program DIFABS) was performed [(8)].

The positions of hydrogen atoms were geometrically generated assuming the appropriate $sp^2$- or $sp^3$-hybridization for the corresponding atoms. Nonhydrogen atoms were refined in the anisotropic but hydrogens — in the isotropic approximations. The final reliability factor R with unit weights was 0.0591 for compound I and 0.0440 for compound III. The highest peak in the final difference map was 0.23 e/Å$^3$ and 0.36 e/Å$^3$ for compounds I and III, respectively.

Table 2. Bond distances (Å) of compounds I and III with e.s.d. values in parentheses

| Bonds | I | III |
|---|---|---|
| | M = Si | M = Ge |
| C(6)—M | 1.951 (10) | 2.011 (5) |
| C(7)—M | 1.842 (11) | 1.935 (7) |
| C(8)—M | 1.861 (14) | 1.936 (8) |
| C(9)—M | 1.829 (11) | 1.926 (5) |
| C(5)—F | 1.330 (10) | 1.355 (5) |
| C(1')—O(1') | 1.399 (11) | 1.412 (6) |
| C(4')—O(1') | 1.412 (12) | 1.417 (7) |
| C(2)—O(2) | 1.213 (11) | 1.215 (5) |
| C(4)—O(4) | 1.223 (13) | 1.221 (7) |
| C(2)—N(1) | 1.397 (13) | 1.386 (7) |
| C(6)—N(1) | 1.400 (9) | 1.407 (5) |
| C(1')—N(1) | 1.475 (12) | 1.478 (6) |
| C(2)—N(3) | 1.368 (13) | 1.370 (7) |
| C(4)—N(3) | 1.362 (11) | 1.372 (6) |
| C(5)—C(4) | 1.453 (15) | 1.446 (8) |
| C(6)—C(5) | 1.330 (13) | 1.332 (7) |
| C(2')—C(1') | 1.492 (12) | 1.516 (6) |
| C(3')—C(2') | 1.516 (19) | 1.520 (11) |
| C(4')—C(3') | 1.492 (16) | 1.490 (10) |

Table 3. Bond angles (deg.) of compounds I and III

| Bond angles | I | III |
|---|---|---|
| | M = Si | M = Ge |
| M—C(6)—N(1) | 122.6 (6) | 123.2 (4) |
| M—C(6)—C(5) | 120.0 (6) | 120.1 (3) |
| C(6)—M—C(7) | 109.9 (5) | 109.8 (3) |
| C(6)—M—C(8) | 111.5 (5) | 111.6 (3) |
| C(6)—M—C(9) | 106.5 (5) | 105.8 (3) |
| C(7)—M—C(8) | 105.8 (6) | 106.6 (3) |
| C(7)—M—C(9) | 110.8 (6) | 111.6 (3) |
| C(8)—M—C(9) | 112.4 (6) | 111.5 (3) |
| F—C(5)—C(4) | 113.6 (8) | 113.6 (4) |
| F—C(5)—C(6) | 121.7 (9) | 120.3 (5) |
| O(1')—C(1')—N(1) | 111.4 (7) | 110.1 (3) |
| O(1')—C(1')—C(2') | 108.8 (8) | 108.2 (4) |
| C(1')—O(1')—C(4') | 109.5 (7) | 109.8 (4) |
| O(1')—C(4')—C(3') | 104.6 (9) | 104.8 (6) |
| O(2)—C(2)—N(1) | 122.6 (9) | 122.9 (5) |
| O(2)—C(2)—N(3) | 123.1 (9) | 121.3 (5) |
| O(4)—C(4)—N(3) | 114.3 (10) | 122.7 (5) |
| O(4)—C(4)—C(5) | 125.5 (8) | 126.2 (4) |
| N(1)—C(2)—N(3) | 114.3 (8) | 115.8 (4) |
| C(2)—N(1)—C(6) | 122.6 (7) | 122.1 (4) |
| C(2)—N(1)—C(1') | 115.2 (7) | 117.6 (3) |
| N(1)—C(6)—C(5) | 117.5 (8) | 116.6 (4) |
| C(6)—N(1)—C(1') | 122.3 (8) | 120.3 (4) |
| N(1)—C(1')—C(2') | 118.6 (10) | 116.2 (4) |
| C(2)—N(3)—C(4) | 128.7 (10) | 127.6 (6) |
| N(3)—C(4)—C(5) | 111.7 (9) | 111.2 (4) |
| C(4)—C(5)—C(6) | 124.7 (8) | 126.2 (4) |
| C(1')—C(2')—C(3') | 102.6 (9) | 101.9 (5) |
| C(2')—C(3')—C(4') | 103.4 (10) | 104.1 (6) |

The intramolecular bond distances for both structures are reported in Table 2, the bond angles in Table 3, selected torsion angles in Table 4.

The final fractional coordinates and equivalent isotropic temperature factors (calculated from refined anisotropic thermal parameters as Beq = $8\pi^2 D_u^{1/3}$, were $D_u$ is the determinant of the $U_{ij}$ matrix) with their esd's in parentheses are listed in Tables 5 and 6.

Table 4. Selective torsion angles (deg.) with e.s.d. in parentheses

| Torsion angles | I | III |
|---|---|---|
| | M = Si | M = Ge |
| N(1)—C(1')—O(1')—C(4') | -123.5 (10) | -121.3 (5) |
| C(3')—C(2')—C(1')—O(1') | 12.3 (14) | 14.3 (7) |
| C(2')—C(1')—O(1')—C(4') | 9.0 (13) | 6.7 (7) |
| C(3')—C(4')—O(1')—C(1') | -26.9 (15) | -25.5 (9) |
| C(4')—C(3')—C(2')—C(1') | -27.5 (15) | -28.8 (8) |
| O(1')—C(4')—C(3')—C(2') | 33.6 (15) | 33.8 (9) |
| C(3')—C(2')—C(1')—N(1) | 140.9 (11) | 138.8 (6) |
| O(2)—C(2)—N(1)—C(6) | 172.0 (7) | 172.8 (4) |
| N(3)—C(2)—N(1)—C(6) | -7.7 (10) | -8.0 (5) |
| M—C(6)—N(1)—C(2) | -173.1 (6) | -173.0 (3) |
| C(5)—C(6)—N(1)—C(2) | 6.8 (10) | 6.6 (5) |
| M—C(6)—N(1)—C(1') | 5.8 (9) | 5.8 (4) |
| C(5)—C(6)—N(1)—C(1') | -174.3 (7) | -174.6 (3) |
| O(1')—C(1')—N(1)—C(2) | 73.2 (9) | 70.8 (5) |
| C(2')—C(1')—N(1)—C(2) | -54.2 (11) | -52.7 (5) |
| O(1')—C(1')—N(1)—C(6) | -105.8 (9) | 108.0 (5) |
| C(2')—C(1')—N(1)—C(6) | 126.8 (10) | 128.5 (4) |
| N(1)—C(2)—N(3)—C(4) | 2.8 (12) | 3.9 (6) |
| C(5)—C(4)—N(3)—C(2) | 2.7 (11) | 1.3 (6) |
| C(6)—C(5)—C(4)—N(3) | -3.9 (11) | -3.0 (6) |
| N(1)—C(6)—C(5)—C(4) | -0.6 (11) | -0.7 (6) |

### *Biological Study*

Macacus rhesus kidney fibroblast cells (FRhK) were cultivated in Eagle's medium supplemented with 30% lactalbumine hydrolysate, 10% bovine serum, 1% HEPES and 2% gentamycin. Ethanol-diluted compounds were added to the incubation medium. The compound concentration was adjusted to 150 mM, solvent concentration — to 0.7%. The control sample was incubated with pure ethanol. $^3$H-Uridine (5 $\mu$Ci/ml, 30 min) or $^3$H-thymidine (5 $\mu$Ci/ml, 60 min) incorporation effectivity was measured to evaluate the intensity of either RNA or DNA biosynthesis. RNA was extracted with LiCl-urea [9], DNA was purified by salting out procedure [10]. Nucleic acid concentration was measured spectrophotometrically, samples were hydrolyzed in 1 M HCl (80°C, 15 min). Radioactivity was measured on the scintillation counter. Measurements were conducted in triplicate. Statistical analysis was carried out by Student's test. Results are presented as mean ± SEM.

Table 5. Fractional coordinates (x $10^4$) for nonhydrogen atoms in compound I

| Atom | x | | y | | z | | Beq | |
|---|---|---|---|---|---|---|---|---|
| Si | 4344 | (3) | 2136 | (2) | 5968 | (1) | 3.48 | (7) |
| F | 5012 | (8) | 1302 | (5) | 4369 | (3) | 5.76 | (25) |
| O(1') | 6541 | (8) | 5413 | (5) | 6581 | (4) | 4.19 | (23) |
| O(2) | 9192 | (8) | 5171 | (6) | 5788 | (4) | 4.09 | (25) |
| O(4) | 7377 | (8) | 2148 | (6) | 3725 | (4) | 4.97 | (27) |
| N(1) | 7235 | (8) | 3727 | (6) | 5890 | (4) | 2.93 | (24) |
| N(3) | 8262 | (11) | 3639 | (7) | 4773 | (5) | 3.28 | (41) |
| C(2) | 8306 | (11) | 4250 | (9) | 5502 | (5) | 3.24 | (43) |
| C(4) | 7270 | (11) | 2633 | (8) | 4366 | (6) | 3.85 | (26) |
| C(5) | 6081 | (11) | 2243 | (8) | 4778 | (5) | 3.94 | (28) |
| C(6) | 6037 | (10) | 2752 | (7) | 5498 | (5) | 3.35 | (23) |
| C(7) | 2362 | (15) | 1367 | (14) | 5067 | (8) | 5.54 | (44) |
| C(8) | 3320 | (16) | 3383 | (10) | 6363 | (9) | 4.64 | (39) |
| C(9) | 5690 | (16) | 1079 | (11) | 6868 | (8) | 4.51 | (43) |
| C(1') | 7432 | (13) | 4290 | (9) | 6736 | (6) | 3.45 | (43) |
| C(2') | 9335 | (16) | 4416 | (12) | 7487 | (7) | 5.18 | (37) |
| C(3') | 9253 | (17) | 5647 | (12) | 7871 | (10) | 6.65 | (39) |
| C(4') | 7832 | (19) | 6309 | (10) | 7086 | (8) | 5.30 | (43) |

Table 6. Fractional coordinates (x $10^4$) for nonhydrogen atoms in compound III

| Atom | x | | y | | z | | Beq | |
|---|---|---|---|---|---|---|---|---|
| Ge | 4304 | (1) | 2097 | (0) | 5970 | (0) | 3.26 | (1) |
| F | 5053 | (5) | 1280 | (3) | 4386 | (2) | 5.50 | (11) |
| O(1') | 6454 | (5) | 5417 | (3) | 6533 | (2) | 4.70 | (9) |
| O(2) | 9141 | (5) | 5177 | (3) | 5755 | (2) | 4.16 | (10) |
| O(4) | 7377 | (5) | 2155 | (3) | 3724 | (2) | 4.85 | (12) |
| N(1) | 7219 | (4) | 3742 | (3) | 5877 | (2) | 2.98 | (9) |
| N(3) | 8261 | (5) | 3648 | (4) | 4765 | (2) | 3.53 | (11) |
| C(2) | 8260 | (6) | 4255 | (4) | 5483 | (3) | 3.33 | (11) |
| C(4) | 7276 | (6) | 2634 | (4) | 4363 | (3) | 3.64 | (11) |
| C(5) | 6120 | (6) | 2249 | (4) | 4784 | (3) | 3.81 | (11) |
| C(6) | 6045 | (5) | 2751 | (3) | 5496 | (3) | 3.10 | (10) |
| C(7) | 2240 | (10) | 1296 | (8) | 5019 | (5) | 5.57 | (17) |
| C(8) | 3253 | (9) | 3367 | (6) | 6404 | (5) | 4.65 | (19) |
| C(9) | 5753 | (9) | 997 | (5) | 6912 | (4) | 4.36 | (15) |
| C(1') | 7349 | (6) | 4288 | (4) | 6714 | (3) | 3.37 | (11) |
| C(2') | 9290 | (7) | 4449 | (6) | 7458 | (3) | 4.44 | (13) |
| C(3') | 9163 | (11) | 5685 | (8) | 7817 | (6) | 6.43 | (24) |
| C(4') | 7734 | (10) | 6323 | (6) | 7029 | (5) | 5.62 | (19) |

**Results and Discussion**

Lithiation of Ftorafur was carried out in THF below –70°C using 4.2 equiv. of lithium diisopropylamide (LDA). After 1 h the resulting clear solution of the C-6 lithiated ftorafur was treated with the corresponding trialkylchlorosilane, -germane or -stannane at –70°C:

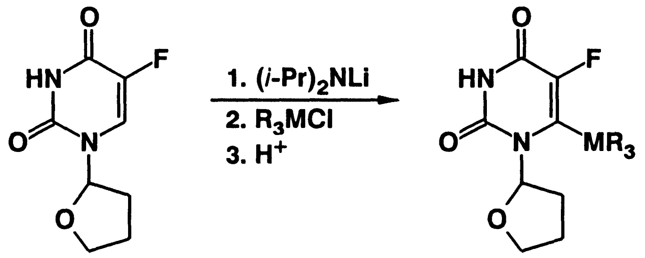

$R_3M$ = $SiMe_3$ (I), $SiEt_3$ (II), $GeMe_3$ (III), $GeEt_3$ (IV), $SnMe_3$ (V)

After working up of the reaction mixture the first 6-substituted organosilicon, -germanium and -tin derivatives of uracil have been obtained.

The 6-substituted organosilicon (I) and organogermanium (III) derivatives of ftorafur are isostructural. The stereoscopic view of the molecule III is given in the Figure 1.

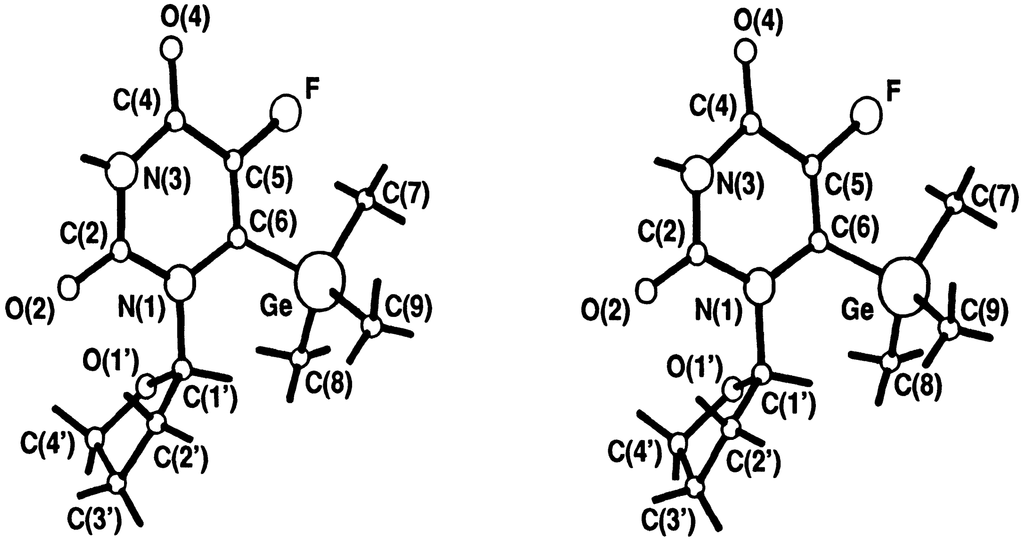

Figure 1. Molecular structure of 1-(2-tetrahydrofuryl)-5-fluoro-6-trimethylgermyluracil III (stereoscopic view)

The bond lengths and angles in uracil and tetrahydrofuranyl (THF) rings of compounds I and III are in agreement with the values obtained in tetrahydrofuranyluracil [11], 1-(2-tetrahydrofuranyl)- [12,13] and 1-(5-trifluoromethyl-2-tetrahydrofuranyl)-5-fluorouracils [14]. The glycosyl bond length in the compounds I and III is slightly shorter than in structures reported in [11–14] and trends to the average value of 1.464 Å [15].

Atoms of the uracil ring in molecules of compounds I and III deviates from the average plane and have screw ($^1S_2$) conformation. Torsional angles (see Table 4) show that the THF rings assume conformations near twist-envelope conformation with atoms C(3') and C(4') out of the plane of the three others by –0.32(2) Å, 0.21(1) Å for I and –0.366(9) Å, 0.156(8) Å for III, respectively. The configuration of the THF ring in both two compounds is C(3')-*exo*—C(4')-*endo*.

In comparison with closely related compounds [(11–14)] the main difference found in reported compounds is the relative orientation of the uracil and five-membered rings. In structures I and III the uracil moiety exhibits a *sin* orientation with respect to the THF ring, while *anti* conformation was found for compounds described in [(11–14)].

Possibly this is a result of the influence of the bulky substituent in 5-fluorouracil ring at C(6). The voluminous $SiMe_3$ and $GeMe_3$ groups make changes in molecular conformations and also in the bond lengths. Inspection of the bond lengths (Table 2) indicates that the C(6) — Si and C(6) — Ge are significantly longer than Si–C and Ge–C bonds with methyl groups carbon.

Similar to crystal structures described in [(12–14)] in the crystal structures of I and III there were found hydrogen bonds N(3)–H(3)...O(2) of an intermolecular nature. Distances between N(3) and O(2) are 2.891 Å and 2.887 Å for compounds I and III, respectively.

A 90 min long incubation with both germyl derivatives III and IV reduced the $^3$H-uridine incorporation rate. Trimethylgermyl derivative (III) caused a two-fold decrease (from 9304.5 ± 3121.5 dpm/μg RNA to 4649 ± 1068.4 dpm/μg RNA), triethylgermyl derivative IV was less effective (to 5433.6 ± 1369.6 dpm/μg RNA) (Fig. 2). The compounds manifested a tendency to reduce the $^3$H-thymidine incorporatiuon, however it was less pronounced and the data were not statistically significant [(16)]. For example, two-hour incubation with compound IV decreased $^3$H-thymidine incorporation from 420.7 ± 130.2 dpm/μg DNA to 307.5 ± 24.75 dpm/μg DNA.

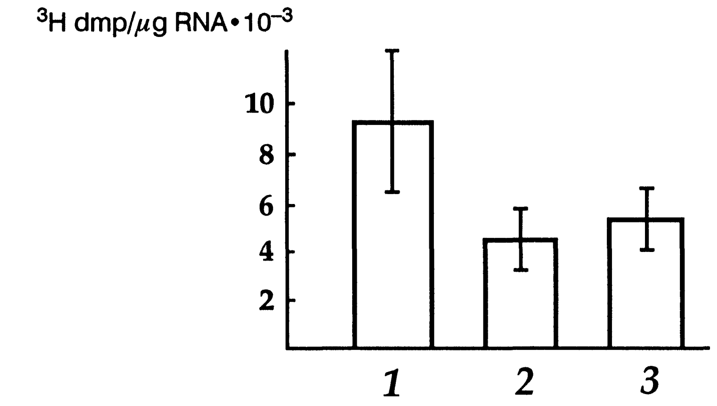

Figure 2. Effects of compounds III and IV on intensity of $^3$H-uridine incorporation. *1* – control (0.7% ethanol); *2* – 150 μM III; *3* – 150 μM IV. Incubation 90 min.

These preliminary results indicate the ability of germanium-modified nucleoside analogues to interfere with transcription and, possibly, with replication processes. Several hypothetical mechanisms of action can be proposed. The substances in question could incorporate in the synthesized molecules and block polymerase movement or act as enzyme inhibitors. Further studies of molecular mechanisms of action are necessary for the evaluation of significance of the germanium-modified compounds as biologically active substances.

### References

1. T. Miyasaka, H. Tanaka, B. Masamori, H. Hayakawa, R. T. Walker, J. Balzarini, E. De Clercq. *J. Med. Chem.* **32** (1989), 2507 (and ref. therein).
2. H. Tanaka, M. Baba, H. Hyakawa, T. Sakamaki, T. Miyasaka, M. Ubasawa, H. Takashima, K. Sekiya, I. Nitta, S. Shigeta, R. T. Walker, J. Balzarini, E.De Clercq. *J. Med. Chem.* **34** (1991), 349.
3. H. Tanaka, M. Baba, S. Saito, T. Miyasaka, H. Takashima, K. Sekiya, M. Ubasawa, I. Nitta, R. T. Walker, H. Nakashima, E. De Clercq. *J. Med. Chem.* **34** (1991), 1508.
4. E. Lukevics, L. Ignatovich, N. Shilina, N. Sjakste, A. Ķemme. *VII-th International Conference on Organometallic and Coordination Chemistry of Germanium, Tin and Lead: Abstracts,* Riga, 1992, 59.
5. E. Lukevics, L. Ignatovich. *Frontiers of organogermanium, -tin and -lead chemistry. VII-th International Conference of Organometallic and Coordination Chemistry of Germanium, Tin and Lead: Proceedings,* Riga, 1993, 331.
6. S. A. Hiller, R. A. Zhuk, M. Yu. Lidak. *Dokl. Acad. Nauk USSR* **176** (1967), 332.
7. G. M. Sheldrick. *SHELX 86, Program for Crystal Structure Solution,* University of Göttingen, (1986).
8. N. Walker, D. Stuart. *Acta Crystallogr.* **A39** (1983), 158.
9. Ch. Auffray, J. Rougeon. *Eur. J. Biochem.* **107** (1980), 303.
10. S. A. Hiller, D. D. Dykes, H. F. Polesky. *Nucl. Acid. Res.* **16** (1988), 1215.
11. C. M. H. Verdegaal, F. B. Martens, C. Romers. *J. Chem. Soc., Perkin Trans. II* (1979), 833.
12. Y. Nakai, K. Yamamotro, K. Terada, T. Uchida, N. Shimuzu, S. Nishigaki. *Chem. Pharm. Bull.* **30** (1982), 2629.
13. G. Dongtao, L. Pinzhe, S. Cheng, W. Fengshan, L. Yuongshen. *Scientia Sinica B* **26** (1983), 1009.
14. A. A. Kemme, Ya. Ya. Bleidelis, M. Yu. Lidak, R. A. Zhuk. *Zhurnal Organicheskoi Khimii* **19** (1983), 1537.
15. R. A. G. de Graaf, G. Admiraal, E. H. Koen, C. Romers. *Acta Crystallogr.* **B33** (1977), 5459.
16. N. Sjakste, D. Meirena, A. Dzene, R. Mežapuķe, L. Ignatovich, E. Lukevics. *Eksperimentine biologija* **3–4** (1992), 26.

**Received: August 3, 1993 - Accepted: September 9, 1993**

